# Brain transcriptome-wide association studies in diverse ancestral populations reveal genes implicated in an anxiety-related phenotype

**DOI:** 10.1093/g3journal/jkaf277

**Published:** 2025-11-17

**Authors:** Maya Z Sharma, Heather E Wheeler

**Affiliations:** Department of Biology, Loyola University Chicago, 1032 W. Sheridan Rd., Chicago, IL 60660, United States; Department of Biology, Loyola University Chicago, 1032 W. Sheridan Rd., Chicago, IL 60660, United States; Program in Bioinformatics, Loyola University Chicago, 1032 W. Sheridan Rd., Chicago, IL 60660, United States

**Keywords:** anxiety, GWAS, TWAS, brain transcriptome, eQTL, colocalization

## Abstract

Anxiety is the most prevalent form of mental illness in the United States. We aimed to identify genetic variation underlying anxiety in diverse ancestral populations through integrating genomic and brain transcriptomic data. We analyzed genome-wide association study (GWAS) summary statistics, using the “Worrier/Anxious Feelings” phenotype from Pan-UK Biobank. We identified 67 independent significant loci in the combined meta-analysis of six ancestral populations (META-GWAS) and 1 locus in the African (AFR) GWAS (*P* < 5.0 × 10^−8^). We performed transcriptome-wide association studies (TWAS) and identified 683 significantly associated genes in the META-TWAS, and 1 gene in the AFR-TWAS (*P* < 3.85 × 10^−6^). Namely, we identified *CADM2* in the META-TWAS and its predicted paralog *SMAGP* in the AFR-TWAS. The genes identified in TWAS were enriched for variants associated with autism, neuroticism, and schizophrenia, highlighting shared genetic architecture among neuropsychiatric traits. In this study, we present these loci and genes as potential targets for future research on anxiety-related phenotypes.

## Introduction

The prevalence of anxiety disorders has surged in recent years. The coronavirus disease-2019 pandemic is a large contributor, as it is estimated that the pandemic led to a 25.6% increase in anxiety disorders worldwide (COVID-19 Mental Disorders Collaborators 2021). Anxiety disorders refer to a group of conditions that enable individuals to experience excessive feelings of fear or worry. Researchers have found that the prevalence of anxiety is higher in women and older adults (Pashazadeh Kan et al. 2021). The most common forms of anxiety disorders are as follows: generalized anxiety disorder, panic disorder, social anxiety disorder, and specific phobias (Konnopka and König 2020). Common physical symptoms are palpitations, dizziness, shortness of breath, and hyperventilation (Shivalkar and Sengupta 2024). Common mental symptoms are difficulty concentrating, persistent feelings of worry, and restlessness (Adwas et al. 2019). We are interested in specifically studying genetic contributors to anxiety-related phenotypes. Heritability estimates of anxiety disorders range from 0.3 to 0.6 (Purves et al. 2020).

Approximately 80% of genome-wide association study (GWAS) published use individuals of primarily European (EUR) descent (Peterson et al. 2019). Genetic associations often fail to replicate across populations due to variations in linkage block patterns and minor allele frequencies because of unique demographic histories (Mogil et al. 2018; Wyss et al. 2018; Peterson et al. 2019; Sirugo et al. 2019; Geoffroy et al. 2020). The Pan-UK Biobank (Pan-UKB) strives to provide GWAS summary statistics across all ancestries included in the UK Biobank, increasing power and the potential for discovery (Karczewski et al. 2025). Due to the evolutionary history of humans, African (AFR) ancestry populations possess millions more single-nucleotide polymorphisms (SNPs) and smaller linkage disequilibrium (LD) blocks compared to EUR or Asian populations (Tishkoff and Williams 2002). Thus, genetic studies in recent AFR ancestry populations have the potential to uncover new underlying mechanisms of disease (Gomez et al. 2014; Pereira et al. 2021; Kachuri et al. 2023).

Transcriptome-wide association studies (TWAS) are powerful tools that combine genetic data with gene expression models to identify gene–trait associations. TWAS enable us to gain deeper insight into how SNPs may regulate gene expression and contribute to the phenotype of interest. PrediXcan is a TWAS method that estimates the component of genetic expression determined by an individual's unique genetics, and then correlates this genetic expression with the phenotype of interest (Gamazon et al. 2015). Summary-PrediXcan (S-PrediXcan) is a method adapted from PrediXcan, which can conduct a TWAS using primarily GWAS summary statistics and gene expression prediction models (Barbeira et al. 2018).

Here, we use GWAS summary statistics of the Pan-UKB phenotype “Worrier/Anxious Feelings” to identify potentially causal genetic associations (Karczewski et al. 2025). Next, we performed TWAS to identify significant correlations between genetically predicted gene expression and the anxiety phenotype. Subsequently, we performed colocalization analyses to determine if the most likely causal SNPs are shared between the GWAS and expression quantitative trait locus (eQTL) signals. Additionally, we performed a gene set enrichment analysis (GSEA) with functional mapping and annotation (FUMA; Watanabe et al. 2017). We conducted a comparative analysis of AFR and META anxiety GWAS results from Pan-UKB to grow our knowledge of potentially causal genetic variants associated with an anxiety-related phenotype.

## Materials and methods

### GWAS summary statistics

The GWAS summary statistics analyzed were obtained from Pan-UKB (Karczewski et al. 2025). Specifically, we used the “Worrier/Anxious feelings” phenotype (*n* = 501,478), which is comprised of individuals of the following genetic ancestries: EUR (*n* = 409,672), Central/South Asian (CSA, *n* = 8,111), AFR (*n* = 6,233), East Asian (EAS, *n* = 2,475), Middle Eastern (MID, *n* = 1,474), and Admixed American (AMR, *n* = 922). This phenotype was collected via a survey, which asked the question “Are you a worrier?” (Karczewski et al. 2025). Cases answered “Yes.” and controls answered “No.” These summary statistics are available for download in the phenotypes section of the Pan-UKB website, listed under phenotype code 1980 (Karczewski et al. 2025).

### Fine-mapping

We performed fine-mapping with LocusZoom to visualize and identify genes located near significant SNPs associated with the Worrier/Anxious Feelings phenotype. LocusZoom is a browser-based tool that can be used to make interactive plots of GWAS summary statistics (Pruim et al. 2010). LocusZoom determines which SNPs are in 95% credible sets through its Bayes' Factor calculations (Wellcome Trust Case Control Consortium et al. 2012). LocusZoom generates Manhattan plots of GWAS summary statistics, labels significant peaks in the data with the nearest gene, and annotates plots with LD statistics, gene tracks, previously identified GWAS associations, and fine-mapping posterior probabilities.

### TWAS

To conduct our TWAS, we began by harmonizing GWAS summary statistics using the 1000G reference panel. We used S-PrediXcan to perform TWAS with inputs of GWAS summary statistics, transcriptome prediction models, and an LD reference, to output genetically predicted gene-level expression association results (Barbeira et al. 2018). We used Multivariate Adaptive Shrinkage in R (MASHR) models as our transcriptome prediction models. MASHR models were utilized because these models perform better or equally as well as other methods across tissues and across populations (Barbeira et al. 2020; Araujo et al. 2023). We used transcriptome prediction models from 13 brain tissues from the Genotype-Tissue Expression (GTEx) Project in our analyses (GTEx Consortium 2013; Barbeira et al. 2020). These brain tissues include cortex, frontal cortex, anterior cingulate cortex, caudate basal ganglia, putamen basal ganglia, nucleus accumbens basal ganglia, hypothalamus, amygdala, hippocampus, cerebellum, cerebellar hemisphere, substantia nigra, and spinal cord (cervical c-1). These specific tissues were selected because they are located in or near the limbic system, the brain's emotional-processing center, and are thought to be linked to anxiety disorders, which may result from imbalances within the limbic system (Martin et al. 2009). After conducting our TWAS, we obtained gene-level expression association results output files. Although TWAS were conducted for all seven cohorts (EUR, CSA, AFR, EAS, MID, AMR, and META), we focus our analysis on the META and AFR cohorts. These two cohorts yielded statistically significant gene associations, making them integral in advancing our understanding of the genetic variation underlying anxiety-related phenotypes in diverse ancestral populations.

### Colocalization

We performed single- and multivariant colocalization using the *coloc* R package, version 5.2.3 (Giambartolomei et al. 2014; Wallace 2021). Single-variant colocalization, *coloc*, employs a statistical method that assumes one causal locus in the region tested for colocalization of GWAS and eQTL signals. Multivariant colocalization, *coloc.susie*, differs from *coloc* as it is a less-stringent method that allows multiple independent loci to associate with the GWAS or gene expression trait (Wallace 2021). We performed colocalization on the top 10 genes identified via TWAS for the META and AFR cohorts. For each gene in the top 10 TWAS results, we compared the GWAS summary statistics of the trait with the GTEx eQTL results of that gene in *coloc.* To perform *coloc.susie*, we used eQTL summary statistics from the GTEx Project that only included individuals of EUR ancestry as our LD reference panel (GTEx Consortium 2013). Colocalization tests five hypotheses: no association with either trait (H_0_), association with trait 1 but not with trait 2 (H_1_), association with trait 2 but not with trait 1 (H_2_), two independent SNP associations with trait 1 and trait 2 (H_3_), and one shared SNP association with trait 1 and trait 2 (H_4_). We defined a significant coloc result as having an H_4_ posterior probability (PP) > 0.5 (Gregga et al. 2023). We defined a significant *coloc.susie* result as any credible set variant with H_4_ PP >0.5. When running *coloc.susie*, we added a window of 500 K bases to the first and last positions of the gene (GTEx Consortium 2013; Nassar et al. 2023). Additionally, when running *coloc.susie*, if the credible sets for the eQTL or the GWAS data were empty (cs = 0), the gene–trait pair was said to have H_4_ and H_3_ probabilities of NA.

### Gene set enrichment analysis (GSEA)

To conduct GSEA, we employed the GENE2FUNC tool available on the FUMA GWAS web application (Watanabe et al. 2017). FUMA serves as a comprehensive tool for annotating, interpreting, and visualizing GWAS results. The GENE2FUNC module allows users to annotate the gene sets in a biological context. To run GENE2FUNC, a list of genes of interest and a list of background genes must be provided and are used in the 2 × 2 enrichment tests. We performed GSEA for our META-TWAS results, as it included more data points and statistically significant findings compared to the AFR-TWAS dataset. Our genes of interest list consisted of 875 genes meeting an FDR threshold of <0.05. The background genes list included all unique genes expressed and tested in the 13 GTEx brain tissues, totaling 18,505 genes. Each query successfully completed provides five different output tabs: Summy of Input Genes, Heatmap, Tissue Specificity, Gene Sets, and a Gene Table. We concentrated our analysis on the gene set results, in which hypergeometric tests are performed to see if the genes of interest are overrepresented in a pre-identified gene set (Watanabe et al. 2017). Specifically, we centered our analysis on GWAS catalog-reported genes, transcription factor (TF) targets (MsigDB c3), cell type signatures (MsigDB c8), and KEGG (MsigDB c2). We focused our analysis on these specific results because they offer valuable insights into biological pathways and contexts in which our genes are potentially involved.

### Multiple testing adjustment

To adjust for multiple testing, we calculated a Bonferroni threshold. The Bonferroni adjustment is a conservative approach that is used to reduce the likelihood we obtain Type I errors (Duggal et al. 2008). This approach adjusts the significance level by dividing 0.05 by the number of tests performed. We used the average number of genes tested per tissue as the denominator in this calculation, and thus our TWAS Bonferroni significance level was *P* < 0.05/13,000 = 3.85 × 10^−6^. In addition to using Bonferroni correction, we also employed a less-stringent approach to adjust for multiple testing, the Benjamini–Hochberg false discovery rate (FDR) adjustment with a 0.05 FDR threshold. We applied the *p.adjust* function in R to perform an FDR adjustment on our *P*-values (Benjamini and Hochberg 1995; Storey 2002). The adjusted *P*-value column present in our data tables is the Benjamini–Hochberg FDR correction for multiple testing (Table 5).

## Results

### Multiple loci associate with the worrier/anxious feelings phenotype in the META cohort

**Fig. 1.**
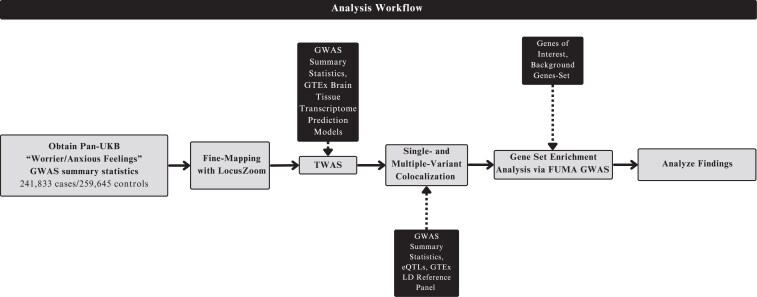
Overview of the analytical workflow. This diagram illustrates the key steps of our analysis. We first obtained GWAS summary statistics for the “Worrier/Anxious Feelings” phenotype from the Pan-UKB and subsequently performed fine-mapping with LocusZoom. Next, we performed TWAS with S-PrediXcan using GTEx brain tissue transcriptome prediction models. Then, we performed colocalization analyses with *coloc* using the summary statistics, eQTLs, and the GTEx LD reference panel. Additionally, we performed a gene set enrichment analysis using the GENE2FUNC tool from the web application, FUMA. Finally, we evaluated and compared significant findings across cohorts.

**Fig. 2.**
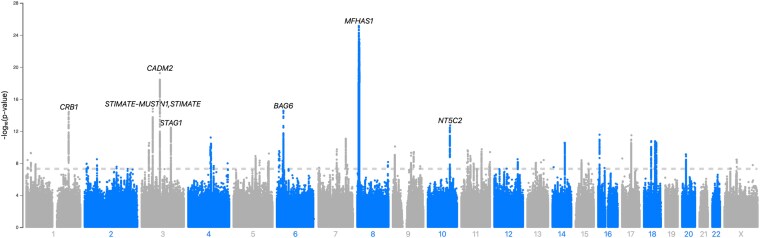
Manhattan plot of GWAS summary statistics of the META pan-UKB worrier/anxious feelings phenotype. This plot displays the GWAS summary statistics for the META cohort. Each data point represents a SNP, with those depicted above the grey dashed line as genome-wide significant (*P* < 5 × 10^−8^).

To identify genomic loci associated with anxiety, we performed several genome- and transcriptome-wide analyses (Fig. 1). We first obtained GWAS summary statistics from the Pan-UKB for the Worrier/Anxious Feelings phenotype (Karczewski et al. 2025). Sample sizes varied across populations, with most of the sample from UKB participants with EUR ancestry (Table 1). We used LocusZoom to visualize and fine-map significant loci (Fig. 2) (Pruim et al. 2010). In total, 8337 SNPs in 67 independent loci significantly associated with the Worrier/Anxious feelings phenotype in the META cohort (*P* < 5 × 10^−8^, Fig. 2). The top hit is in the Multifunctional ROCO Family Signaling Regulator 1, *MFHAS1* gene (Table 2).

**Table 1.** Ancestral population distribution of cases and controls in worrier/anxious feelings phenotype.

Cohort	Cases (%)	Controls (%)	Total
EUR	231,995 (57)	177,677 (43)	409,672
CSA	4,462 (55)	3,649 (45)	8,111
AFR	2,740 (44)	3,493 (56)	6,233
EAS	1,214 (49)	1,261 (51)	2,475
MID	914 (62)	560 (38)	1,474
AMR	508 (55)	421 (45)	929
META	241,833 (48)	259,645 (52)	501,478

Distribution of cases and controls in each cohort within the Pan-UKB dataset, including European (EUR), Central/South Asian (CSA), African (AFR), East Asian (EAS), Middle Eastern (MID), Admixed American (AMR), and the META cohort. The META cohort combines the results from each individual ancestral population’s GWAS in a meta-analysis. The values in the parentheses are percentages that indicate the proportion of cases and controls within each respective population.

**Table 2.** Top 10 pan-UKB META loci identified via fine-mapping.

Nearest Gene(s)	rsID	Chr	Pos	Ref	Alt	Effect Size	*P*-value	95% Credible Set	Posterior Probability	Total SNPs in Credible Set
*MFHAS1*	rs7013471	8	8,687,325	A	G	0.051	7.2×10^−26^	TRUE	0.219	16
*CADM2*	rs62250713	3	85,513,793	A	G	0.046	5.8 × 10^−20^	TRUE	0.099	439
*STIMATE-MUSTN1,STIMATE*	rs6798941	3	52,893,465	C	T	−0.042	1.6 × 10^−15^	TRUE	0.254	64
*BAG6*	rs36173461	6	31,610,189	TAAAG	T	0.038	2.8 × 10^−15^	TRUE	0.320	5
*CRB1*	rs78108344	1	197,388,870	C	T	−0.046	3.9 × 10^−15^	TRUE	0.353	6
*NT5C2*	—	10	104,908,360	CA	C	−0.037	1.9 × 10^−13^	TRUE	0.410	75
*STAG1*	rs6439649	3	136,371,691	G	T	0.036	3.8 × 10^−13^	TRUE	0.051	46
*RBFOX1*	rs55997507	16	7,666,664	G	C	0.035	2.8×10^−12^	TRUE	0.279	24
*LRRC37A2, ARL17A*	rs199913382	17	44,625,866	A	C	0.043	3.2×10^−12^	TRUE	0.586	12
*LINC02503*	—	4	105,023,666	CA	C	−0.042	6.2 × 10^−12^	TRUE	0.316	50

This table highlights the most significant SNPs identified via fine-mapping of the Pan-UKB META cohort with LocusZoom. The alternate allele is the effect allele.

### One locus significantly associates with the worrier/anxious feelings phenotype in the AFR cohort

**Fig. 3.**
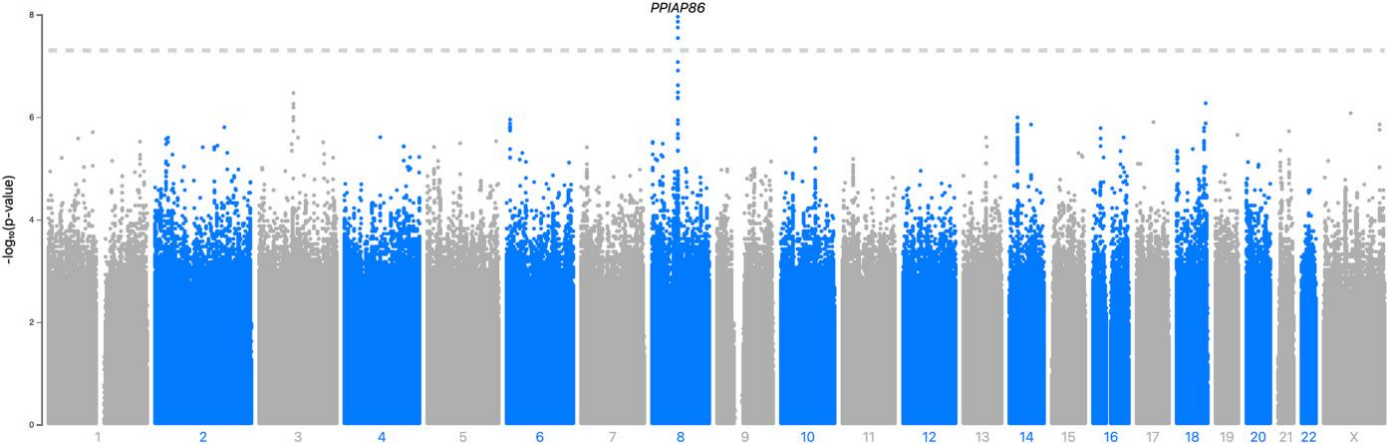
Manhattan plot of GWAS summary statistics of the AFR-pan-UKB worrier/anxious feelings phenotype data. This plot illustrates the GWAS summary statistics for the AFR population. Each data point represents a SNP, with those depicted above the grey dashed line as genome-wide significant (P < 5 × 10^−8^).

We also determined if any new loci were discovered when only those with AFR ancestries were included in the GWAS (Fig. 3). In total, four SNPs significantly associated with the Worrier/Anxious feelings phenotype in the AFR cohort (*P* < 5 × 10^−8^, Fig. 3). The top hit, rs151103418, is located on chromosome 8, at position 66,265,578, its reference allele is cytosine, and its alternate/effect allele is thymine, with an effect size of 0.42 (*P* = 1.1 × 10^−8^). This hit is in the 95% credible set. This hit is located upstream of the Peptidylprolyl Isomerase A Pseudogene 86, *PPIAP86,* gene. The probability that rs151103418 at this locus is the causal SNP is 0.312. The alternate allele frequency of this SNP is 0.077 in AFR ancestral populations, and it is not present in non-AFR ancestral populations and thus would not be discovered in the META analysis of predominantly EUR ancestral populations (Ensembl release 112) (Harrison et al. 2024).

### Genetically regulated gene expression in brain tissues associates with the worrier/anxious feelings phenotype in META and AFR cohorts

**Fig. 4.**
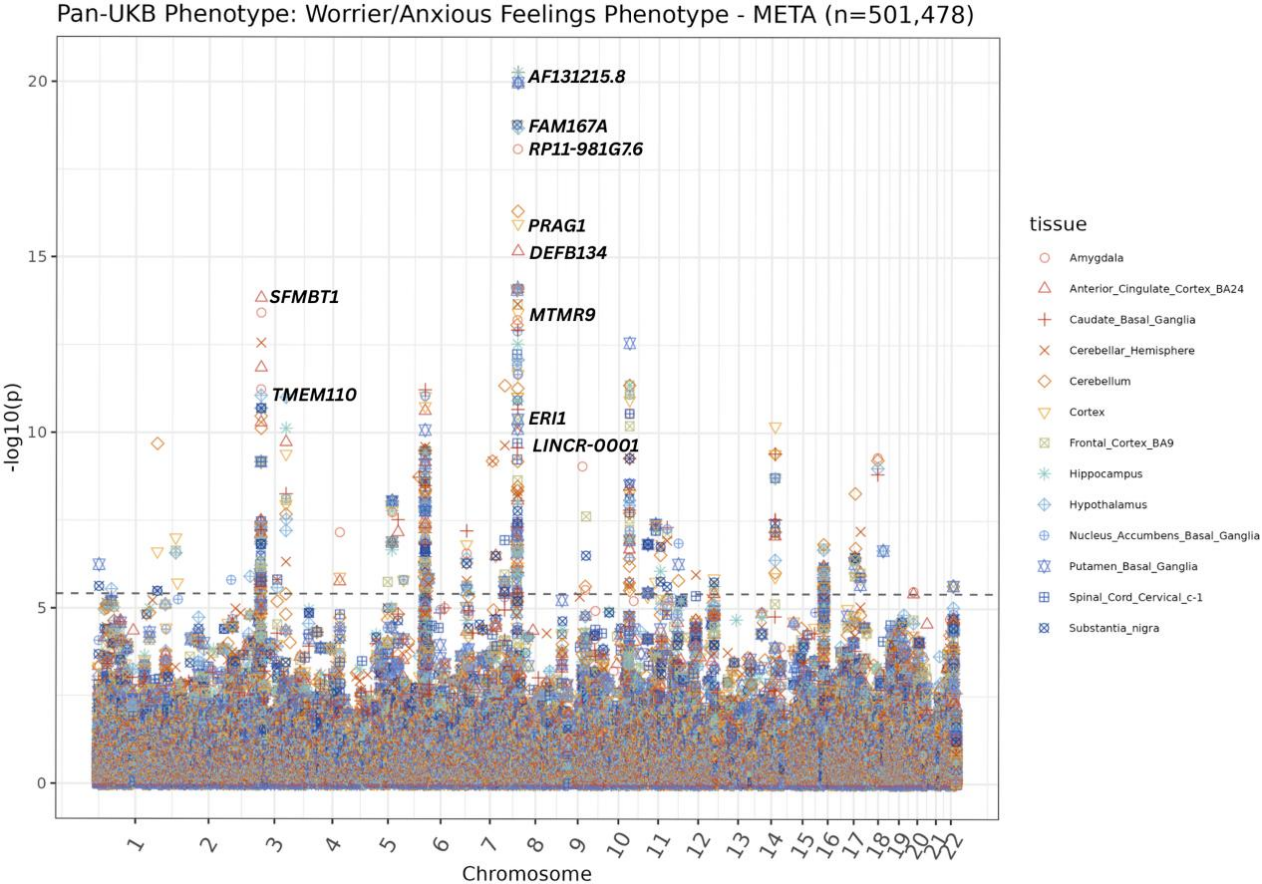
Manhattan plot of META-TWAS results. This plot illustrates the META-TWAS results, with each data point representing a gene. The distinct color and shape of the data point corresponds to the tissue in which the gene expression prediction model was trained. The grey dashed line marks the threshold for genome-wide significance (*P* < 3.85×10^−6^). Data points above this line are considered statistically significant and may indicate important gene-trait associations.

To better understand the genetic components underlying our phenotypes of interest, we performed TWAS. This approach integrates GWAS summary statistics and expression models to identify predicted gene expression associations with the phenotype of interest. In our META-TWAS, we identified 683 genes associated with the anxiety phenotype (*P* < 3.85 × 10^−6^) in various brain tissues (Supplementary Table 1, Fig. 4). The top 10 META-TWAS results are depicted in Table 3. The top hit in the META-TWAS results was the *AFR131215.8* gene (*P* = 5.31×10^−21^) with the prediction model trained in hippocampus tissue. Decreased expression of *AFR131215.8* is also significantly associated with anxiety in models trained in caudate basal ganglia, hypothalamus, nucleus accumbens basal ganglia, anterior cingulate cortex, putamen basal ganglia, and cerebellum tissues. The next most significant gene in the META-TWAS was Peak Related Kinase-Activating Pseudokinase 1 (*PRAG1)* using transcriptome prediction models trained in cortex and substantia nigra tissues (Table 3). Additionally, our META-TWAS found that decreased expression levels of *CADM2* in hypothalamus and spinal cord tissues are potentially correlated to the incidence of the Worrier/Anxious Feelings phenotype, as evidenced by a negative effect size (*P* = 1.0 × 10^−4^, Supplementary Table 1).

**Table 3.** Top 10 unique META-TWAS hits.

Gene	Chr	Pos	Gene Name	Z-Score	Effect Size	*P*-value	Tissue
ENSG00000270076	8	11,201,144	*AF131215.8*	−9.4	−0.81	5.3 × 10^−21^	Hippocampus, Caudate Basal Ganglia, Hypothalamus, Nucleus Accumbens Basal Ganglia, Anterior Cingulate Cortex, Putamen Basal Ganglia, Cerebellum
ENSG00000275342	8	8,317,736	*PRAG1*	−9.0	−0.61	1.7 × 10^−19^	Cortex, Substantia Nigra
ENSG00000154319	8	11,421,476	*FAM167A*	9.0	0.25	2.1 × 10^−19^	Hypothalamus, Cortex, Amygdala
ENSG00000272505	8	10,486,806	*RP11-981G7.6*	−8.9	−0.15	8.2 × 10^−19^	Amygdala
ENSG00000205882	8	1,193,174	*DEFB134*	−8.1	−0.15	6.8 × 10^−16^	Anterior Cingulate Cortex
ENSG00000253641	8	10,474,565	*LINCR-0001*	−7.8	−0.098	7.0 × 10^−15^	Caudate Basal Ganglia, Hypothalamus
ENSG00000104643	8	11,284,416	*MTMR9*	−7.8	−0.53	7.9 × 10^−15^	Cortex, Amygdala, Anterior Cingulate Cortex, Hippocampus, Caudate Basal Ganglia, Spinal Cord Cervical (C-1), Nucleus Accumbens Basal Ganglia, Cerebellum, Hypothalamus
ENSG00000104626	8	9,002,147	*ERI1*	7.8	0.17	8.6 × 10^−15^	Putamen Basal Ganglia
ENSG00000163935	3	52,903,572	*SFMBT1*	−7.7	−0.11	1.5 × 10^−14^	Anterior Cingulate Cortex
ENSG00000213533	3	52,836,219	*TMEM110*	−7.7	−0.13	1.7 × 10^−14^	Frontal Cortex

This table depicts the top 10 unique loci identified with TWAS in the META cohort. We used GRCh38 build to identify the chromosome and position of each gene. The most significant Z-score, effect size, and *P*-value is shown for the first tissue listed for each gene, but all tissues listed met the Bonferroni-adjusted significance threshold of *P* < 3.85 × 10^−6^.

**Fig. 5.**
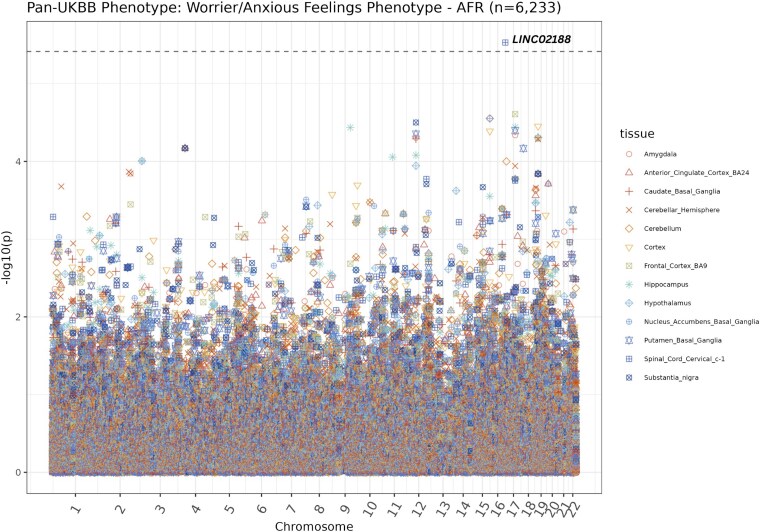
Manhattan plot of AFR-TWAS results. This plot illustrates the AFR-TWAS results, with each data point representing a gene. The distinct color and shape of the data point corresponds to the tissue in which the gene expression prediction model was trained. The grey dashed line marks the threshold for genome-wide significance (*P* < 3.85×10^−6^). Data points above this line are considered statistically significant and may indicate important gene-trait associations.

In our AFR-TWAS, we identified one statistically significant gene (*P* < 3.85 × 10^−6^) (Fig. 5, Supplementary Table 2). Increased predicted expression of the top hit, *LINC02188*, associated with anxiety using models trained in spinal cord cervical tissue (*P* = 3.0 × 10^−6^). The top 10 AFR-TWAS results are depicted in Table 4.

**Table 4.** Top 10 unique AFR loci identified using TWAS.

Gene	Chr	Pos	Gene Name	Z-score	Effect Size	*P*-value	Tissue
ENSG00000261175	16	86,710,122	*LINC02188*	4.7	3.8	3.0 × 10^−6^	Spinal Cord Cervical
ENSG00000150527	14	39,230,231	*CTAGE5*	−4.6	−0.6	4.4 × 10^−6^	Cerebellar Hemisphere
ENSG00000108846	17	50,634,777	*ABCC3*	−4.2	−0.5	2.5 × 10^−5^	Frontal Cortex, Hippocampus, Putamen Basal Ganglia, Amygdala
ENSG00000095906	16	1,782,932	*NUBP2*	−4.2	−3.9	2.8 × 10^−5^	Nucleus Accumbens Basal Ganglia, Cerebellum, Cortex
ENSG00000170545	12	51,244,558	*SMAGP*	−4.2	−0.6	3.2 × 10^−5^	Substantia Nigra, Putamen Basal Ganglia, Amygdala, Caudate Basal Ganglia, Hippocampus
ENSG00000179284	19	12,965,159	*DAND5*	−4.1	−0.9	3.5 × 10^−5^	Cortex, Caudate Basal Ganglia, Hypothalamus, Frontal Cortex, Cerebellar Hemisphere
ENSG00000197816	9	97,307,304	*CCDC180*	4.1	6.0	3.7 × 10^−5^	Hippocampus
ENSG00000174145	4	37,448,181	*NWD2*	4.0	27.7	6.8 × 10^−5^	Frontal Cortex, Hippocampus, Hypothalamus, Amygdala, Cerebellar Hemisphere, Substantia Nigra, Anterior Cingulate Cortex
ENSG00000134278	18	12,606,464	*SPIRE1*	−4.0	−3.9	6.8 × 10^−5^	Putamen Basal Ganglia
ENSG00000186652	11	57,389,932	*PRG2*	−3.9	−2.0	8.8 × 10^−5^	Hippocampus

This table depicts the top 10 unique loci identified with TWAS in the AFR cohort. We used GRCh38 build to identify the chromosome and position of each gene. The most significant Z-score, effect size, and *P*-value is shown for the first tissue listed for each gene.

### Colocalization analyses identify *CTAGE5* gene as colocalized in AFR cohort

To further validate our TWAS results, we performed single-variant colocalization on the top 10 Pan-UKB META-TWAS hits and subsequently performed multivariant colocalization (Supplementary Table 3) (Al-Barghouthi et al. 2022). Colocalization allows for the distinction between results that are potentially confounded by LD and provides evidence that these SNPs are functioning via gene expression regulation to influence anxiety risk (Hormozdiari et al. 2016; Barbeira et al. 2018). Analysis of the single- and multivariant colocalization results for the META-TWAS genes did not identify any genes with an H_4_ value that met our significance threshold for colocalization of H_4_ PP > 0.5 (see Methods).

**Fig. 6.**
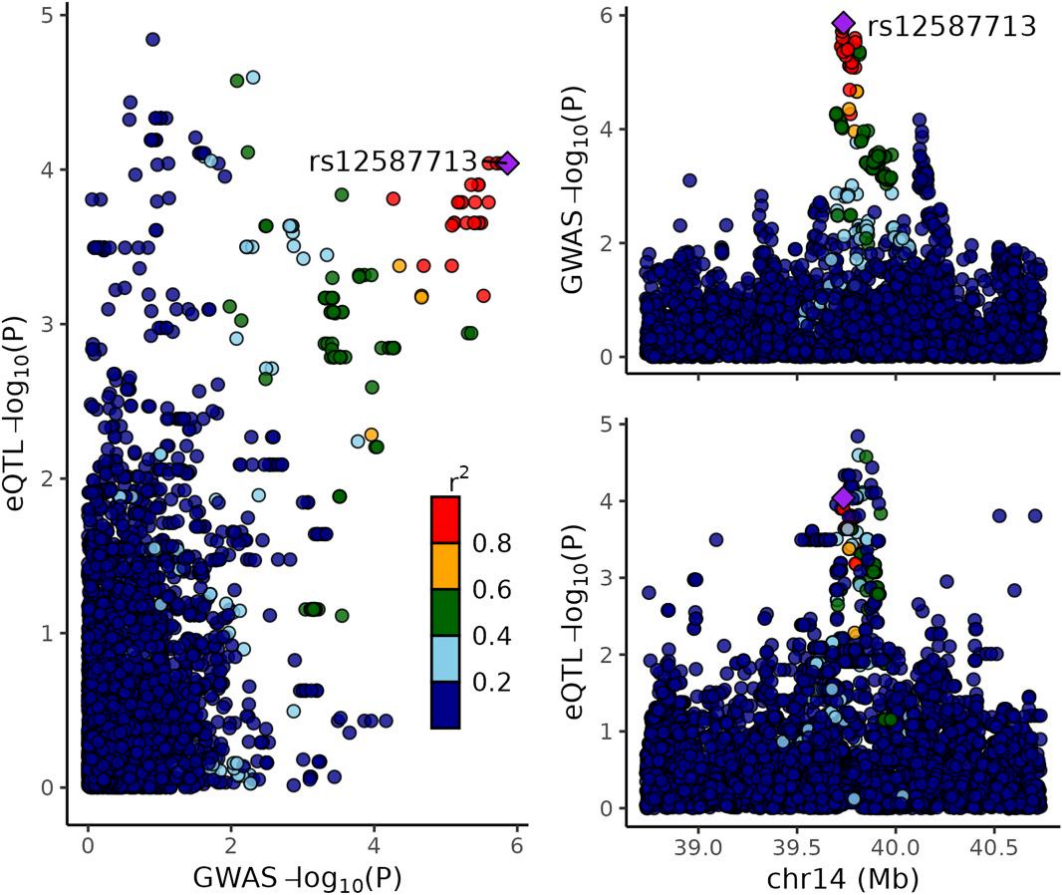
LocusCompareR plot of *CTAGE5* displaying single-variant colocalization between GWAS and eQTL signals in cerebellar hemisphere tissue with AFR LD reference panel. The left panel shows a scatter plot of −log_10_(*P*-values) for GWAS and eQTL associations, with rs12587113 highlighted. The right panel displays two plots for the GWAS (top right) and eQTL (bottom right) data across chromosome 14. The overlapping peaks evident in the GWAS and eQTL data are suggestive of shared genetic architecture (H_4_ PP = 0.79).

Subsequently, we performed single- and multivariant colocalization on the top 10 AFR-TWAS hits (Supplementary Table 4). Analysis of the single-variant AFR colocalization results identified *CTAGE5* present in cerebellar hemisphere tissue as the only gene that met our significance threshold of an H_4_ PP > 0.5, with an H_4_ PP of 0.79. We used the LocusCompareR package to visualize this GWAS-eQTL colocalization (Fig. 6; Liu et al. 2019). Multivariant colocalization analysis in the AFR cohort identified only one credible set in *CTAGE5* as colocalized with an H_4_ PP of 0.74.

### GSEA of META-TWAS highlights biological pathways and gene sets enriched with genes of interest

We conducted GSEAs to investigate whether our top META-TWAS genes are enriched in specific biological pathways and processes. We focused on key outputs, including GWAS catalog-reported genes, TF targets, cell type signatures, and KEGG pathway tables generated by FUMA (Watanabe et al. 2017).

In the GWAS catalog-reported genes, we identified 108 gene sets as enriched in our META-TWAS results (Supplementary Table 5). The top 10 gene set results consist of neurological-related phenotypes, namely, autism spectrum disorder/schizophrenia, asthma and major depressive disorder, neuroticism, feeling worry, and brain morphology (Table 5). The “Feeling worry” gene set enrichment (*P* = 6.38×10^−20^) closely relates to our phenotype of interest, Worrier/Anxious feelings. Additionally, top TWAS genes were enriched in gene sets related to neuroticism, which is characterized by a predisposition to negative emotional states, including anxiety, depression, anger, and emotional instability (Widiger and Oltmanns 2017).

**Table 5.** GWAS catalog reported genes identified via GENE2FUNC FUMA GWAS tool.

Gene Set	*N*	*n*	*P*-value	Adjusted *P*-Value
Autism spectrum disorder or schizophrenia	417	125	2.25 × 10^−64^	9.95 × 10^−61^
Asthma and major depressive disorder	60	36	5.94 × 10^−32^	1.31 × 10^−28^
General factor of neuroticism	62	31	2.24 × 10^−24^	3.31 × 10^−21^
Neuroticism	120	41	6.54 × 10^−24^	7.24 × 10^−21^
Schizophrenia	567	89	1.06 × 10^−22^	9.39 × 10^−20^
Brain morphology (MOSTest)	957	117	2.69 × 10^−20^	1.99 × 10^−17^
Feeling worry	41	23	6.38 × 10^−20^	4.03 × 10^−17^
Waist-hip index	495	77	1.74 × 10^−19^	9.64 × 10^−17^
Waist-to-hip ratio adjusted for BMI	677	92	4.83 × 10^−19^	2.38 × 10^−16^
A body shape index	341	56	1.74 × 10^−15^	7.69 × 10^−13^

This table presents the top 10 gene sets enriched in the META-TWAS dataset. N represents the number of genes in the gene set, and *n* indicates the number of genes overlapping between our query list and the genes in the gene set. Supplementary Table 5 depicts the full result table and specific gene names. The adjusted *P*-value is the Benjamini-Hochberg FDR.

In the TF targets analysis, 14 gene sets were significantly enriched in our META-TWAS gene associations (FDR < 0.05), and the most enriched TF binding site was NFY_Q6_01 (*P* = 6.45 × 10^−6^; Supplementary Table 5). In the cell type signatures analysis, two gene sets were identified as enriched, including MANNO_MIDBRAIN_NEUROTYPES_HSERT (*P* = 5.85 × 10^−5^) and MANNO_MIDBRAIN_NEUROTYPES_HGABA (*P* = 9.43 × 10^−5^; Supplementary Table 5). In the KEGG analysis, 10 gene sets were enriched: Allograft Rejection, Graft Versus Host Disease, Antigen Processing and Presentation, Type I Diabetes Mellitus, Asthma, Thyroid Disease, Cell Adhesion Molecules (CAMs), Systemic Lupus Erythematosus, Viral Myocarditis, and Leishmania Infection (Supplementary Table 5). The KEGG_CELL_ADHESION_MOLECULES_CAMS gene set includes *CADM2* (*P* = 9.21 × 10^−5^).

Some of the genes identified via FUMA we previously identified as the most significantly associated with anxiety in our META-GWAS and META-TWAS results. BAG Cochaperone 6 (*BAG6)* and *CADM2* were in our top 10 META-GWAS results (Table 2). FUMA identified *CADM2* to be enriched in two GWAS catalog gene sets, neuroticism and feeling worry (Supplementary Table 5). It is likely that the “feeling worry” enrichment includes overlapping UK Biobank data analyzed here, and as a result, this enrichment is expected.

## Discussion

Through the integration of fine-mapping, TWAS, colocalization, and GSEA, we identified several key genes that may contribute to the Worrier/Anxious Feelings phenotype from Pan-UKB.

Fine-mapping of the META cohort highlighted *CADM2* among the top 10 hits. *CADM2* has previously been associated with psychological traits and obesity (Morris et al. 2019). *Cadm2* regulates body weight and energy homeostasis in mice (Yan et al. 2018). Sanchez-Roige et al. (2023) found that *Cadm2* knockout mice display impulsive personality traits and were more likely to engage in risk-taking behavior, which is consistent with our findings.

Our META-TWAS results supported this result, showing that decreased expression of *CADM2* and *PRAG1* is potentially correlated to the incidence of the Worrier/Anxious Feelings phenotype (Supplementary Table 4). Loci within *PRAG1* influence selective serotonin reuptake inhibitors and neuroticism personality in patients with depression (Amare et al. 2018). Additionally, morphogenesis of glial cells has been discussed as a close link between anxiety and relief behavior. A particular signaling cascade of interest is the Rnd2/Prag1/Fyn cascade. Induced knockdown of *Rnd2* or *Prag1* decreases Fyn phosphorylation, which impacts the oligodendroglial cell differentiation and myelination (Fukatsu et al. 2023). Studies show that when oligodendrocytes or myelin are impacted, anxiety-like behaviors can occur or increase (Edgar and Sibille 2012; Chen et al. 2015; Zuo et al. 2024). Knockout of *Prag1* potentially leading to anxiety is supported by our results, as META-TWAS showed a negative effect size for *PRAG1,* meaning decreased levels of *PRAG1* are associated with increased likelihood of anxiety (Table 3).

We compared the AFR and META association results and identified *SMAGP* and *CADM2* as predicted paralogs, with both genes contributing to cell adhesion (Stelzer et al. 2016; Hu et al. 2022). A potential paralogous relationship may suggest a potential convergent mechanism through which CAMs may modulate anxiety-related phenotypes across populations (Koonin 2005). *SMAGP,* Small Cell Adhesion Glycoprotein, was amongst the top 10 AFR-TWAS results, while *CADM2* was identified via fine-mapping of the META cohort GWAS summary statistics. Additionally, our META-TWAS found that decreased expression levels of *CADM2* in hypothalamus and spinal cord tissues are potentially correlated to the incidence of the Worrier/Anxious Feelings phenotype, as evidenced by a negative effect size (*P* = 1.0 × 10^−4^, Supplementary Table 1). *CADM2* specifically encodes a member of the synaptic cell adhesion molecule 1 (SynCAM) family, which belongs to the immunoglobulin (Ig) superfamily (Pasman et al. 2022). Similarly, *SMAGP* functions in cell adhesion as it contributes to epithelial cell adhesion. Additionally, *SMAGP* contains binding domains for protein 4.1 and the PDZ domain of MAGUK proteins (Jia et al. 2018). In our TWAS, *SMAGP* levels predicted from substantia nigra tissues had an effect size of −0.6, thus decreased expression of the *SMAGP* gene is potentially correlated to the incidence of the Worrier/Anxious Feelings Phenotype (Table 4). If *SMAGP* is functioning similarly to *CADM2*, this finding supports those of Sanchez-Roige et al. (2023) in which they found *Cadm2* knockout mice displayed impulsive personality traits and were more likely to engage in risk-taking behavior. Pasman et al. (2022) demonstrated that *CADM2* is involved in various psycho-behavioral traits, suggesting it may serve as a common biological denominator across multiple traits.

Our colocalization analysis further identified *CTAGE5* in cerebellar hemisphere tissue as significant in the AFR cohort. Prior work demonstrated that cTAGE5 forms a complex with MEA6 and is critical for brain development, suggesting a potential role in neurodevelopmental contributions to anxiety (Zhang et al. 2018).

GSEA provided additional context by highlighting enrichment of gene sets related to autism spectrum disorder and schizophrenia (Adjusted *P* = 9.95 × 10^–61^; Table 5). Schizophrenia is often comorbid with anxiety, with the prevalence of anxiety disorder significantly higher in patients with schizophrenia (45%) compared to controls (16%; Kiran and Chaudhury 2016). Approximately 40% of individuals with autism spectrum disorder are diagnosed with one form of anxiety (van Steensel et al. 2011; Zaboski and Storch 2018). The second most enriched gene set is asthma and major depressive disorder (Table 5). Research has found that individuals with asthma are three times more likely to develop internalizing disorders (depression, anxiety, OCD, etc.; Caulfield 2021). Research has shown that major depressive disorder and anxiety share many symptoms, often occur concurrently, and that individuals experiencing both conditions tend to have worsened symptoms and greater chronicity (Kessler et al. 2008; Hofmeijer-Sevink et al. 2012).

Cell-type enrichment analyses implicated midbrain-associated gene sets, including MANNO_MIDBRAIN_NEUROTYPES_HSERT and MANNO_MIDBRAIN_NEUROTYPES_HGABA (*P* = 9.43 × 10^−5^) (Supplementary Table 5). The midbrain plays a central role in motor control and emotion (Smidt et al. 2003). The dopaminergic system, located in the midbrain, is vital in the regulation of motivation (DeGroot et al. 2020). Additionally, dopamine has been shown to have a “modulating” effect in anxiety-like behavior, with increased dopamine levels contributing to increased anxiety (Zarrindast and Khakpai 2015). *BAG6* is in six GWAS catalog gene sets, and *BAG6* has been implicated in contributing to mechanisms underlying severe mental illness and cardiometabolic disease (Supplementary Table 5; Hayman et al. 2023).

We compared our TWAS results to a recently published large-scale meta-analysis across four biobanks, including UK Biobank, and two psychiatric cohorts (Friligkou et al. 2024). None of our Bonferroni significant genes also met Bonferroni significance in Friligkou et al. (2024). This could be due, in part, to the use of different transcriptome prediction models between the two studies: MASHR models here and FUSION and MultiXcan models in Friligkou et al. (2024). Future work could investigate the performance of different transcriptome prediction models across these cohorts.

A limitation of our study is that the phenotype is based on self-reported survey data rather than clinical diagnoses. While survey-based assessments can offer valuable insights, individual variability in survey interpretation may lead to less-precise phenotype classification. Future research should incorporate clinically validated diagnostic criteria to strengthen and confirm our findings. Another limitation is the fact that our TWAS and colocalization analysis lacked ancestry-matched transcriptome and LD reference panels, potentially impacting the accuracy of causal SNP associations. Future studies should incorporate ancestry-matched reference panels to correct this limitation.

This study provides new insights into potentially causal genetic factors underlying Pan-UKB's Worrier/Anxious Feelings Phenotype, by identifying several candidate genes and pathways that may serve as future potential biomarkers or therapeutic targets. This study focused on diverse ancestral populations as it is vital that research is conducted with individuals of diverse ancestry because our unique ancestries are critical factors in the foundation of our genetic architecture (Wang and Gazal 2023 Oct 21).

## Supplementary Material

jkaf277_Supplementary_Data

## Data Availability

All GWAS summary statistics are publicly available from the Pan-UK Biobank (https://pan.ukbb.broadinstitute.org/phenotypes) at https://pan-ukb-us-east-1.s3.amazonaws.com/sumstats_flat_files/categorical-1980-both_sexes-1980.tsv.bgz. Code is available at https://zenodo.org/records/15594914. Supplemental material available at G3 online.
