# Supplementary material for: Brain transcriptome-wide association studies in diverse ancestral populations reveal genes implicated in an anxiety-related phenotype
Source: G3 (Bethesda). 2025 Nov 17;16(1):jkaf277. doi: 10.1093/g3journal/jkaf277 (PMC12774604; doi:10.1093/g3journal/jkaf277)
Supplement: jkaf277_Supplementary_Data [file jkaf277_supplementary_data.zip › Supplemental_Material_Legends_G3-2025-406354.docx]

**Supplemental Material Legends**

***Table S1. Summary statistics from META-analysis TWAS Using GTEx Brain Tissues***

This file contains the META-analysis TWAS summary statistics for the “Worrier/Anxious Feelings” phenotype from the Pan-UKB dataset, conducted using S-PrediXcan across 13 brain tissues from GTEx. Variables include gene, gene_name, UK_zscore, UK_beta, UK_pvalue, UK_var_g, UK_best_gwas_p, UK_largest_weight, tissue, and UK_pvalue_fdr.

***Table S2. Summary statistics from AFR- ancestry cohort TWAS Using GTEx Brain Tissues***

This file contains the AFR-ancestry TWAS summary statistics for the “Worrier/Anxious Feelings” phenotype from the Pan-UKB dataset, conducted using S-PrediXcan across 13 brain tissues from GTEx. Variables include gene, gene_name, UK_zscore, UK_beta, UK_pvalue, UK_var_g, UK_best_gwas_p, UK_largest_weight, tissue, and UK_pvalue_fdr.

***Table S3. Colocalization analysis results (coloc and coloc.susie) of META-analysis data***

This file contains colocalization analysis results based on the META cohort of the “Worrier/Anxious Feelings” phenotype. Analyses were performed using both the standard *coloc* and *coloc.susie,* which accounts for multiple causal variants within a locus. The input data included GWAS summary statistics and tissue-specific eQTLs from GTEx.

***Table S4. Colocalization analysis results (coloc and coloc.susie) of AFR-ancestry data***

This file contains colocalization analysis results based on the AFR cohort of the “Worrier/Anxious Feelings” phenotype. Analyses were performed using both the standard *coloc* and *coloc.susie,* which accounts for multiple causal variants within a locus. The input data included GWAS summary statistics and tissue-specific eQTLs from GTEx.

***Table S5. META Gene Set Enrichment Analysis Results from FUMA***

This file contains the results of a gene set enrichment analysis conducted using FUMA on the genes we identified through the META-analysis of the “Worrier/Anxious Feelings” phenotype. Genes were selected for analysis if the met an FDR threshold of p<0.05. The results include multiple tables depicting categories of enriched gene sets, such as GWAS catalog reported genes, TF targets, Cell type signatures, and KEGG.
